# Refractory Hemorrhagic Radiation Proctitis Managed With Sequential Endoscopic, Topical, and Hyperbaric Oxygen Therapy

**DOI:** 10.7759/cureus.111495

**Published:** 2026-06-25

**Authors:** Taha Shakarchi

**Affiliations:** 1 Department of Internal Medicine, Beth Israel Lahey Health, Burlington, USA

**Keywords:** argon plasma coagulation, chronic radiation proctitis, hyperbaric oxygen therapy, prostate cancer radiation, radiofrequency ablation, rectal bleeding, sucralfate enema

## Abstract

Chronic radiation proctitis is a recognized late complication of pelvic radiation therapy and may present with rectal bleeding, urgency, altered bowel habits, ulceration, and transfusion-requiring anemia. Management can be challenging when bleeding persists despite standard endoscopic therapy. We present the case of a 72-year-old man with a history of prostate cancer treated with radiation therapy in 2016 who developed refractory hemorrhagic radiation proctitis in 2023. He initially underwent three sessions of argon plasma coagulation (APC) for rectal bleeding but was later hospitalized with acute blood loss anemia requiring transfusion. Colonoscopy demonstrated post-treatment rectal ulcers with visible vessels, which were treated with bipolar electrocautery. He subsequently received compounded sucralfate enemas with improvement in bleeding, followed by repeat endoscopic treatment of residual radiation proctitis with APC. Due to persistent symptoms, mesalamine suppositories and rectal radiofrequency ablation were used. Ongoing bleeding ultimately prompted referral for hyperbaric oxygen therapy, with completion of 30 sessions over seven weeks and resolution of rectal bleeding. Follow-up flexible sigmoidoscopy showed resolution of radiation proctitis with one clean-based rectal ulcer. Biopsies were negative for cytomegalovirus (CMV), herpes simplex virus (HSV), and dysplasia. This case highlights the stepwise and often multimodal management required for refractory chronic radiation proctitis and supports consideration of hyperbaric oxygen therapy when bleeding persists despite topical and endoscopic interventions.

## Introduction

Radiation proctitis is an inflammatory and ischemic injury of the rectal mucosa that may occur after radiation therapy for pelvic malignancies, including prostate, cervical, bladder, and rectal cancers [1,2]. Acute radiation proctitis typically occurs during or shortly after radiation therapy and is often self-limited. Chronic radiation proctitis may develop months to years after exposure and is characterized by progressive mucosal injury, telangiectasias, friability, ulceration, fibrosis, strictures, and, in severe cases, fistula formation [1].

Rectal bleeding is one of the most common and clinically significant presentations of chronic radiation proctitis. In mild cases, conservative and topical therapies may be sufficient. However, patients with persistent or severe bleeding may require endoscopic therapy such as argon plasma coagulation (APC), bipolar cautery, or radiofrequency ablation [1,3,4]. Hyperbaric oxygen therapy is another treatment option for refractory disease, particularly when bleeding or ulceration persists despite conventional treatment [1,5]. We report a case of refractory hemorrhagic radiation proctitis requiring sequential topical, endoscopic, and hyperbaric oxygen-based therapy. This case was previously presented as a poster titled “Refractory Radiation Proctitis Toolkit: The Usual and Beyond!” at the American College of Gastroenterology Annual Scientific Meeting in Philadelphia, Pennsylvania, in October 2024.

## Case presentation

A 72-year-old man with a history of hypertension, hyperlipidemia, and prostate cancer treated with radiation therapy in 2016 presented in 2023 with recurrent rectal bleeding. His symptoms were attributed to chronic radiation proctitis. Given persistent bleeding, he initially underwent three sessions of thermal ablation with APC.

Two weeks after the third APC session, in October 2023, he was hospitalized with acute blood loss anemia requiring blood transfusion. Colonoscopy demonstrated three post-treatment rectal ulcers with visible vessels. These were successfully treated with bipolar electrocautery. Given concern for ongoing mucosal injury and recurrent bleeding, he was treated with compounded sucralfate enemas, 2 g in 20 mL of water twice daily for 30 days, with subsequent improvement in bleeding.

Repeat colonoscopy showed a few clean-based post-APC ulcers, as well as residual radiation proctitis. The residual radiation proctitis was treated with additional APC. He was then started on mesalamine suppositories twice daily. Due to improved but persistent bleeding, he underwent rectal radiofrequency ablation on January 8, 2024.

Despite these interventions, he continued to experience intermittent rectal bleeding. He subsequently underwent 30 sessions of hyperbaric oxygen therapy over seven weeks. After completing hyperbaric oxygen therapy, his rectal bleeding resolved. Follow-up flexible sigmoidoscopy demonstrated resolution of radiation proctitis with one residual clean-based rectal ulcer. Biopsies from the ulcer were negative for cytomegalovirus (CMV), herpes simplex virus (HSV), and dysplasia. The ulcer was managed expectantly with planned follow-up to confirm healing. Mesalamine suppositories were eventually discontinued.

**Figure 1:**
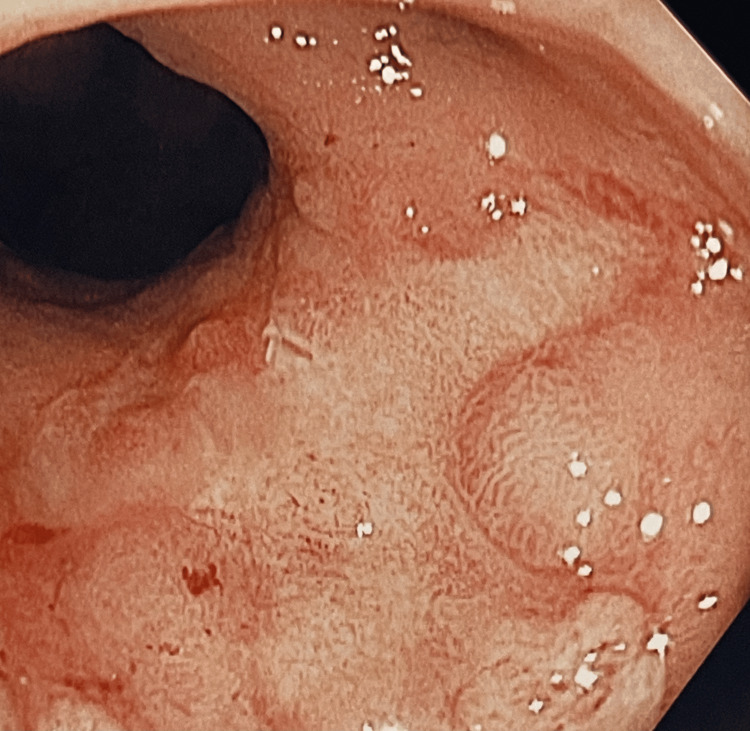
Endoscopic appearance of chronic radiation proctitis with friable mucosa and radiation-associated vascular ectasia. Written informed consent was obtained from the patient for publication of this de-identified case report and accompanying endoscopic images.

**Figure 2:**
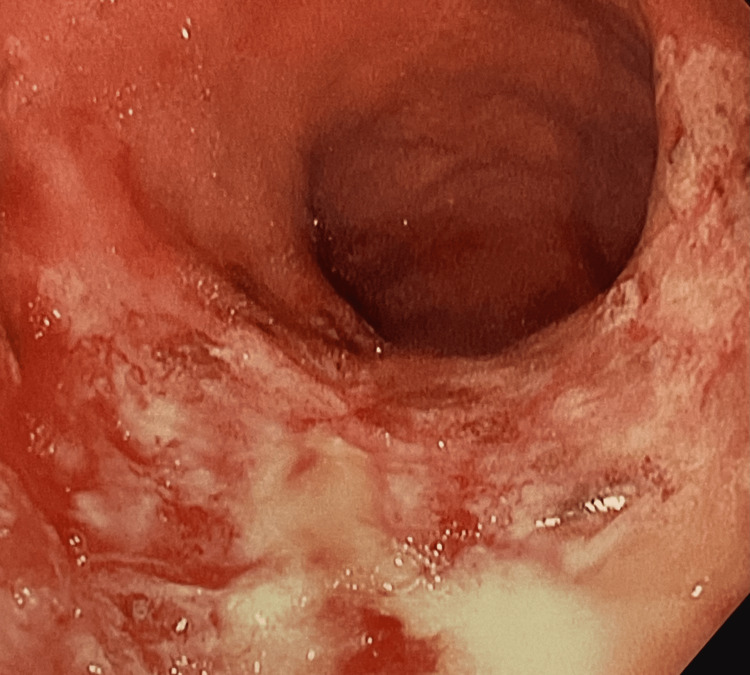
Post-treatment rectal ulceration after argon plasma coagulation with erythematous and friable surrounding mucosa. Written informed consent was obtained from the patient for publication of this de-identified case report and accompanying endoscopic images.

**Figure 3:**
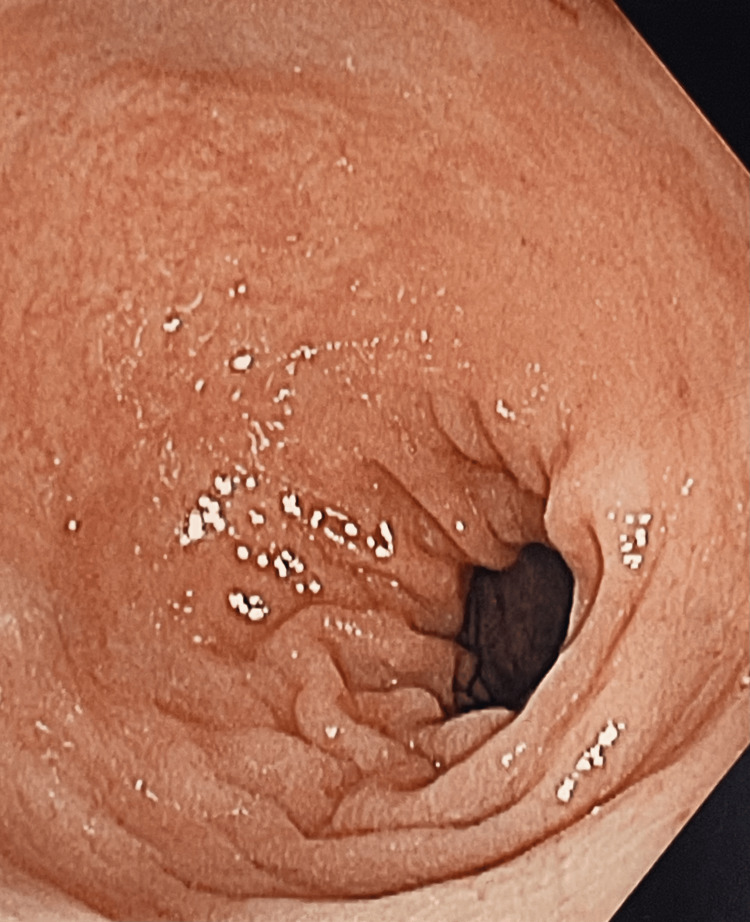
Follow-up flexible sigmoidoscopy after multimodal therapy demonstrating marked interval mucosal healing. Multimodal therapy included topical therapy, radiofrequency ablation, and hyperbaric oxygen therapy. Written informed consent was obtained from the patient for publication of this de-identified case report and accompanying endoscopic images.

Representative endoscopic images demonstrated friable radiation-associated vascular ectasia (Figure 1), post-treatment ulceration after thermal therapy (Figure 2), and subsequent interval mucosal healing after multimodal treatment including radiofrequency ablation and hyperbaric oxygen therapy (Figure 3).

## Discussion

Chronic radiation proctitis can be difficult to manage because the underlying injury is not limited to superficial inflammation. Radiation-induced endarteritis, ischemia, fibrosis, and impaired mucosal healing contribute to mucosal friability, telangiectasia formation, bleeding, and ulceration. The clinical spectrum ranges from mild rectal bleeding and bowel habit changes to severe hemorrhage, transfusion-dependent anemia, strictures, ulceration, and fistulas [1,6,7].

Endoscopy plays a central role in both diagnosis and management. In this patient, endoscopic evaluation confirmed hemorrhagic radiation proctitis and later identified post-treatment ulceration with visible vessels. APC is commonly used for bleeding radiation proctitis because it allows non-contact coagulation of superficial telangiectasias [1,3,8,9]. However, repeated thermal therapy can be associated with ulceration, particularly in previously irradiated tissue with impaired healing. This patient developed post-treatment ulcers after serial APC, including visible vessels requiring bipolar electrocautery.

Topical sucralfate enemas were used as part of the treatment sequence and were associated with improvement in bleeding. Sucralfate may provide mucosal protection and promote local healing in radiation-injured tissue. Mesalamine suppositories were also attempted, although the evidence supporting mesalamine in chronic radiation proctitis is less robust than for other therapies. In this case, mesalamine was used as an adjunctive therapy rather than a definitive treatment.

Radiofrequency ablation was pursued because of persistent bleeding despite previous topical and endoscopic therapy. Endoscopic treatment options for bleeding chronic radiation proctitis include APC, bipolar electrocoagulation, heater probe therapy, and radiofrequency ablation [4,10]. Compared with deeper thermal injury, radiofrequency ablation may provide more controlled superficial ablation, although available data remain limited.

Hyperbaric oxygen therapy was ultimately used after ongoing bleeding despite multiple interventions. Hyperbaric oxygen may improve tissue oxygenation, stimulate angiogenesis, and support healing in chronically irradiated tissue [1,5]. In this patient, completion of 30 sessions over seven weeks was followed by resolution of rectal bleeding and near-complete endoscopic healing. This supports the role of hyperbaric oxygen therapy as an important option for refractory chronic radiation proctitis, particularly when bleeding persists despite topical and endoscopic management. Practical barriers include access, cost, insurance authorization, and the need for repeated treatment sessions.

This case is notable because the patient required nearly the full therapeutic toolkit for refractory hemorrhagic radiation proctitis, including APC, bipolar electrocautery, sucralfate enemas, mesalamine suppositories, radiofrequency ablation, and hyperbaric oxygen therapy. The case also illustrates the importance of repeated reassessment, because persistent bleeding after treatment may reflect ongoing radiation proctitis, post-ablation ulceration, visible vessel bleeding, infection, dysplasia, or malignancy. In this patient, biopsies were negative for CMV, HSV, and dysplasia, supporting continued expectant management of the residual clean-based ulcer.

## Conclusions

Refractory hemorrhagic radiation proctitis may require sequential and multimodal therapy. This case demonstrates the limitations of repeated thermal therapy alone and highlights the value of individualized treatment using topical therapy, endoscopic hemostasis, radiofrequency ablation, and hyperbaric oxygen therapy. Hyperbaric oxygen therapy should be considered in patients with persistent bleeding or impaired mucosal healing despite conventional endoscopic and medical interventions.
